# Henoch–Schonlein purpura due to sodium valproate: A case report

**DOI:** 10.1002/ccr3.5596

**Published:** 2022-03-22

**Authors:** Firozeh Hosseini, Mojgan Safari

**Affiliations:** ^1^ 48430 Hamadan University of Medical Sciences Hamadan Iran

**Keywords:** anticonvulsive, Henoch–Schonlein Purpura (HSP), purpura, sodium valproate

## Abstract

We report a 5‐year‐old girl with a generalized seizure treated with sodium valproate syrup. She returned to the clinic with Henoch–Schonlein purpura after 20 days due to the consumption of sodium valproate. To our knowledge, this is the second case of sodium valproate‐induced HSP.

## Abstract 2

Consumption of sodium valproate can cause Henoch–Schonlein purpura.

## 1 INTRODUCTION

Henoch–Schonlein Purpura (HSP) is an acute systemic immunoglobulin A (IgA)‐mediated vasculitis. It involves the small vessels of the skin, the gastrointestinal tract, the joints, and the kidneys. The central nervous system (CNS) and lungs are involved rarely. It mostly affects patients in the age range of 2–11 years.^1^ The male‐to‐female ratio is 1.5.^2^ The cause of HSP is unknown, but most patients are affected by antecedent upper respiratory tract infection. Currently, drug‐induced HSP is also reported. The immune system seems to play a role in blood vessels involvement.^3^ The abnormal immune response to drugs and infections may be a factor in HSP.^4^ The diagnosis of HSP is based on clinical presentations, including the presence of petechial or palpable purport with a special pattern in lower limbs and buttock in addition to one or more of the following signs: abdominal pain, arthritis or arthralgia, and renal involvement.^5^ Among the drugs suspected to the development of HSP are antibiotics,^6^ anti‐emetics,^7^ analgesics,^8^ anticoagulants,^9^ and anticonvulsant drugs,^10^ TNF‐α inhibitors for autoimmune diseases,^11^ antihypertensive drugs,^12^ antineoplastic,^13^ anti‐arrhythmic agents,^14^ and antidepressants.^15^ However, anticonvulsant drugs are rarely reported with HSP.^9^ What we report is possibly the youngest case with HSP that was developed following sodium valproate therapy. This indicates the potential adverse effects of sodium valproate in children.

## 2 CASE REPORT

A 5‐year‐old girl was admitted to our neurology clinic with multiple episodes of upward gaze, blinking, and falling started 6 months ago. She had not any perinatal insult and her parents declared language delay, language disability, and mild motor delay. In her past medical history, she had an admission for head trauma a year ago without any seizure or loss of consciousness. In electroencephalography, numerous bursts of sharp and spike‐wave in various epochs were observed. Brain MRI reported to be normal. Sodium valproate prescribed for the patient with the initial dose of 5 mg/kg which increased to 15 mg/kg after 4 days. The patient was admitted again after 22 days with diffuse petechial rash in lower limbs and ecchymotic lesion on the buttock with necrotizing areas without fever and abdominal pain but 4 days before that, she had presented severe abdominal pain that healed without treatment. At first, sodium valproate tablet was replaced by carbamazepine. During 4 days of admission, the patient recovered without abdominal pain and hematuria. Abdominal sonography was normal. In laboratory test: WBC = 5500 (P = 78%, L = 18%), HB = 14 g/dl, HCT = 40%, PLT = 362,000, PT = 12 s, INR = 1, PTT = 32 s, ESR = 12 mm, CRP = Neg, AST = 33 IU/L, ALT = 27 IU/L, BUN = 13 mg/dl, Cr = 0/6 mg/dl, U/A = normal, LDH = 298, Alkaline phosphatase = 299 IU/L, ANA = Neg, and RF = Neg. Echocardiography was normal. The patient was discharged with good condition and prescribed with prednisolone tablets 0.5 mg/kg. Four days after, the patient returned to the hospital with severe abdominal pain, dysentery, hematuria, and frequent seizure episodes. The prednisolone tablet was discontinued by parents. In the physical examination, skin rashes continued to exist. Abdominal sonography ruled out intussusception. Laboratory tests were similar to first admission, except the urine analysis that presented hematuria. Methylprednisolone administered at 1 mg/kg and carbamazepine replaced with clobazam tablet and levetiracetam syrup. After 5 days, the patient was discharged with urinalysis follow‐up and prescribed with 1‐week prednisolone and pantoprazole tablets. In a later follow‐up, skin eruption disappeared, and the patient had no seizure with levitiracetam and clobazam. Diagnosis of Henoch–Schonlein purpura was made on the basis of the presence of skin rashes as petechia and ecchymosis, abdominal pain, GI bleeding, hematuria, and normal coagulation tests.

## 3 DISCUSSION

Henoch–Schonlein purpura (HSP) is the most common vasculitis in children. The incidence of HSP is 8–20 children per 100,000 annually. HSP is an IgA‐mediated small‐vessel vasculitis. IgA deposition in vessel walls of the skin, joints, intestines, and kidneys leads to symptoms in these organs. It occurs more frequently in children than in adults. Rashes with small bruises over the legs or buttock are the main symptom. In general, HSP has a good prognosis. Renal or CNS involvement worsens the prognosis. HSP often occurs following an acute infection, which provides strong evidence of infectious trigger. Recently, drug‐induced HSP has been reported by many authors. Previously, however, drugs such as ciprofloxacin, cocaine, acetylsalicylic acid, acetyl cholinesterase, carbidopa/levodopa inhibitors, carbamazepine, streptokinase, cefuroxime, etanercept, diclofenac, vancomycin, and clarithromycin have been involved in the induction of HSP.^16^ Anticonvulsant drugs are rarely reported in the list of drugs associated with HSP.^9^ However, many anticonvulsive drugs have serious skin eruption side‐effects such as Steven–Johnson syndrome and toxic epidermal necrolysis.^17^ Sodium valproate is an anticonvulsive drug with a rare serious skin eruption. It is alternatively used for patients with anticonvulsant hypersensitivity syndrome.^18^ The first case of sodium valproate‐induced HSP in children was reported by Koumaki et al. in 2015 in a 10‐year‐old patient that consumed sodium valproate due to absence seizure. In the literature review, we found only a few anticonvulsive drugs (e.g., carbamazepine and levetiracetam) in association with HSP and the current patient seems to be the second case of sodium valproate‐induced HSP. Most drug‐induced HSP cases are reported in adults, but more reports have been published recently in children. In this report we presented a 5‐year‐old girl who developed HSP during treatment with sodium valproate. The patient recovered and the anticonvulsive drug was switched to clobazam. The combination of skin rashes as petechia and ecchymosis, abdominal pain, GI bleeding, hematuria, and normal coagulation tests proves the diagnosis of Henoch–Schonlein purpura.

## 4 CONCLUSION

Although rarely, sodium valproate can induce HSP which have to be considered in children with seizure.

## CONFLICT OF INTEREST

The authors do not have any conflict of interest.

## AUTHOR CONTRIBUTIONS

All the authors have contributed equally to conception, design, manuscript preparation, critical revision, and finalization. All the authors agree to be accountable for all aspects of the work.

## ETHICAL APPROVAL

Ethical approval was not required for the publication of this report.

## CONSENT

Written informed consent was obtained from the father of the patient to publish this report in accordance with the journal's patient consent policy.

## Data Availability

Data are available in Besat Hospital, Hamadan, Iran.

## References

[1] Gardner‐Medwin JM , Dolezalova P , Cummins C , Southwood TR . Incidence of Henoch‐schonlein Purpura, Kawasaki disease, and rare vasculitidides in children of different ethic origins. Lancet. 2002;360:1197‐1292. 12401245 10.1016/S0140-6736(02)11279-7

[2] Bluman J , Goldman RD . Henoch‐Schönlein purpura in children. Can Fam Physician. 2014;60(11):1007‐1010. 25551129 PMC4229160

[3] Tizard EJ , Hamilton‐Ayres MJJ . Henoch‐Schönlein purpura. Arch Dis Child Educ Pract Ed. 2008;93:1‐8. 18208978 10.1136/adc.2004.066035

[4] Sohagia AB , Gunturu SG , Tong TR , Hertan HI . Henoch‐Schonlein purpura—a case report and review of the literature. Gastroenterol Res Pract. 2010;2010:1‐7. 10.1155/2010/597648 PMC2874920 20508739

[5] Ozen S , Ruperto N , Dillon MJ , et al. EULAR/PReS endorsed consensus criteria for the classification of childhood vasculitides. Ann Rheum Dis. 2006;65(7):936‐941. 16322081 10.1136/ard.2005.046300 PMC1798210

[6] ‐ Wakefield IR , Hunter DA . Antibiotic associated Henoch‐Schonlein purpura syndrome. Br J Clin Pract. 1988;42(12):525‐526. 3256349

[7] Upputuri S , Prasad S . Metoclopramide induced delayed non‐hrombocytopenic purpuric rash. Clin Drug Investig. 2006;6(12):745‐747. 10.2165/00044011-200626120-00008 17274681

[8] Santoro D , Stella M , Castellino S . Henoch‐ Schönlein purpura associated with acetaminophen and codeine. Clin Nephrol. 2006;66(2):131‐134. 16939070 10.5414/cnp66131

[9] Borrás‐Blasco J , Girona E , Navarro‐Ruiz A , et al. Acenocoumarol‐induced Henoch‐Schönlein purpura. Ann Pharmacother. 2004;38(2):261‐264. 14742763 10.1345/aph.1D270

[10] Kaneko K , Igarashi J , Suzuki Y , Niijima S , Ishimoto K , Yabuta K . Carbamazepine induced thrombocytopenia and leucopenia complicated by Henoch‐Schönlein purpura symptoms. Eur J Pediatr. 1993;152(9):769‐770. 8223813 10.1007/BF01953999

[11] Asahina A , Ohshima N , Nakayama H , et al. Henoch‐Schönlein purpura in a patient with rheumatoid arthritis receiving etanercept. Eur J Dermatol. 2010;4:521‐522. 10.1684/ejd.2010.0977 20413370

[12] Moots RJ , Keeling PJ , Morgan SH . Adult Schönlein‐Henoch purpura after enalapril. Lancet. 1992;340(8814):304‐305. 10.1016/0140-6736(92)92391-r 1353212

[13] Aktas B , Topcuoglu P , Kurt OK , et al. Severe Henoch‐Schönlein purpura induced by cytarabine. Ann Pharmacother. 2009;43(4):792‐793. 19336650 10.1345/aph.1L608

[14] Kuo M , Winiarski N , Garella S . Nonthrombocytopenic purpura associated sequentially with nifedipine and diltiazem. Ann Pharmacother. 1992;26(9):1089‐1090. 1421671 10.1177/106002809202600908

[15] Hong KL , Hsiang Chuo Y , Han Chen C , Hsiang HY . Henoch‐Scholein purpura related to fluoxetine medication in a child. Neuropsychiatry. 2018;8(5):1649‐1651.

[16] Duvuru G , Stone JH . Henoch‐Schönlein purpura. In: Imboden JB , Hellmann DB , Stone JH , eds. Current Diagnosis and Treatment. Rheumatology, 3rd edn. McGraw‐Hill; 2013.

[17] Inada A , Oyama S , Niinomi I , Wakabayashi T , Iwanaga K , Hosohata K . Association of Stevens‐Johnson syndrome and toxic epidermal necrolysis with antiepileptic drugs in pediatric patients: subgroup analysis based on a Japanese spontaneous database. J Clin Pharm Ther. 2019;44(5):775‐779. 31231846 10.1111/jcpt.13001

[18] Ashrafi MR , Heidari M . General principles of the medical management of epilepsy in children: a literature review. Rev Clin Med. 2018;5(2):49‐53.

